# Staff Perception of Respect for Human Rights of Users and Organizational Well-being: A Study in Four Different Countries of the Mediterranean Area

**DOI:** 10.2174/1745017902016010109

**Published:** 2020-07-30

**Authors:** Yosra Zgueb, Antonio Preti, Alessandra Perra, Sofian El-Astal, Cesar Ivan Aviles Gonzalez, Martina Piras, Giorgia Testa, Iskren Kirolov, Giorgio Tamburini, Uta Ouali, Goce Kalcev, Ferdinando Romano, Viviane Kovess, Mauro Giovanni Carta

**Affiliations:** 1 Department of Psychiatry A, Razi Hospital, La Manouba, Tunisia; 2 Faculty of Medicine of Tunis, University of Tunis El Manar, Manouba, Tunisia; 3 Center for Liaison Psychiatry and Psychosomatics, University Hospital, University of Cagliari, Cagliari, Italy; 4 Department of Psychology, Al Azhar University-Gaza, Gaza, Palestine; 5 Department of innovation Sciences and Technologies, University of Cagliari, Cagliari, Italy; 6 Department of Medical Sciences and Public Health, University of Cagliari,Cagliari, Italy; 7 Department of Higher Education,European Association of Institutions in Higher Education, Brussels, Belgium; 8 University Goce Delcev, Stip, North Macedonia; 9 Department of Public Health and Infectious Diseases, Sapienza University of Rome, Rome, Italy; 10 Laboratoire de Psychopathologie et Processus de Santé, Université Paris-Descartes, Paris, France

**Keywords:** Human rights, Job satisfaction, Wellbeing, Mental health, Psychiatry, Mediterranean area, Multicenter study

## Abstract

**Background:**

The perception by mental health service staff of respect for users' rights is a fundamental component of organizational well-being. The objective of this work is to examine how cultural differences and the working context can influence the perception of respect for users' rights in mental health professionals in the Mediterranean area.

**Methods:**

An observational survey carried out in four different mental health networks in four countries of the Mediterranean area (Tunisia, North-Macedonia, Italy, Palestine). Each invited participant fulfilled a format on socio-demographic information and coded the Well-Being at Work and Respect Right Questionnaire (WWRR). All data were encrypted and analysed using the Statistical Package for Social Sciences (SPSS) version 20. The Games-Howell post-hoc test was used to assess differences between countries. The Games-Howell test does not assume equal variances and sample sizes. Eta-squared (η^2^) was used as a measure of effect size in the ANOVA (η^2^ around 0.01, 0.06, and 0.14 are considered small, medium, and large, respectively).

**Results:**

The sample included 590 professionals working in the mental health field. The four countries showed statistically significant differences with regards to the quality rights assessment tool. Participants from Italy reported, on average, the highest scores across the questions. There were also differences across the countries about the perception of the impact of available resources on the effectiveness of care (η2 = 0.106).

**Conclusion:**

Our findings offer a useful insight into the perception of the quality of mental health services, especially from a users’ rights point of view.

## 1 INTRODUCTION

The perception by mental health services staff of the respect for users' rights is a fundamental component of organizational well-being, as previously described in the literature [1-3]. Mental health is a field often criticized for not respecting the rights of users. However, young mental health professionals now live in an environment increasingly sensitive towards these issues [4-5]. Indeed, the relevance of the rights of people with disabilities was underlined by the United Nations Convention on the Rights of Persons with Disabilities [6], and the theme specifically dealing with the rights of psychosocial disabilities has been recently placed on public attention worldwide by the WHO Quality Rights initiative [7].

The importance of the respect of users' rights by the mental health services staff is also confirmed by the validation study, at least in the Mediterranean area, of the Questionnaire on Well-Being at Work and Respect of Rights (WWRR) which is reported by another work of this same special issue [8].

The objective of this work is to examine, through the administration of this same questionnaire in different Mediterranean countries, how cultural differences and work context can influence the perception of respect for users' rights in mental health professionals.

## 2 METHODS

### 2.1 Design

This is an observational study survey carried out in four different mental health networks in four countries of the Mediterranean area (Tunisia, North-Macedonia, Italy, Palestine).

### 2.2 Setting

In Tunisia, the survey was carried out at « Razi » University Hospital, the only psychiatric hospital in the country. The Razi Hospital is located in the southwestern suburbs of Tunis. Razi includes seven adult inpatient units, one adult outpatient unit, one unit for child and adolescent psychiatry, and one forensic psychiatry unit. The staff of non-clinical services such as laboratories and pharmacy were also included in the sample.

In North Macedonia, surveys and interviews were carried out on staff of the outpatient facilities of the Psychiatric private centers and the patient facilities of Psychiatric counseling and psychotherap units, in Skopje.

In South Sardinia (Italy), the study was carried out in the “Department of Mental Health ASL8”. This is a community health care public service covering an area of around 500,000 adult inhabitants including the southwest of Sardinia and the city of Cagliari. It includes 6 units of community care and two hospital wards of 15 beds, each located at the general hospital “Santissima Trinità”.

In the Gaza Strip (Palestine), the survey was carried out in the mental health centers belonging to the Palestinian Ministry of Health and in the centers of the Gaza Community Mental Health Program (GCMHP) in Gaza City, Deir al-Balah and Khan Younis.

The four samples come from not perfectly homogeneous networks but each somewhat representative of mental healthcare systems in the country of origin.

### 2.3 Sample

A random sample (1/3) representative of staff of the mental health care network in the four different above described care networks in Italy, Tunisia, North-Macedonia, and Gaza Strip (Palestine) was invited to take part in the study.

### 2.4 Instruments

Each selected study participant completed a format on socio-demographic information and coded the Well-Being at Work and Respect Right Questionnaire (WWRR), a seven-item questionnaire. The first five items use a Likert scale from 1: “Not satisfied at all” to 6 “Totally satisfied”. In item 6, the Likert scale runs in the opposite direction; however, for the sake of simplicity, the results will be illustrated in the same direction as the other five items. The seventh item uses a nominal scale choice. The questionnaire asks about the perception of job satisfaction of health professionals, users’ satisfaction of healthcare provided, satisfaction of organizational aspects of work, professional perception of the respect of users and staff human rights, adequacy of resources and beliefs about specific needs for professionals.

### 2.5 Ethics

The Institutional Review Board of the University Hospital of Cagliari, Italy, approved the protocol of the study, which has been conducted in accordance with the guidelines of the 1995 Declaration of Helsinki and its revisions [9].

### 2.6 Statistics

All data were coded and analyzed using the Statistical Package for Social Sciences (SPSS) version 20. Additional analyses were carried out in R [10]. All tests were two-tailed. The significance threshold was set at p < 0.05.

Means with standard deviations were reported for continuous variables. Counts and percentages were reported for categorical variables. The reliability of the questionnaire was measured by Cronbach’s alpha. For group comparisons, reliability values of 0.70 are considered satisfactory [11].

Analysis of Variance (ANOVA) was used to compare the groups of participants by country. The Games-Howell posthoc test was used to assess differences between countries. The Games-Howell test does not assume equal variances and sample sizes. *Eta*-*squared* (η^2^) was used as a measure of effect size in the ANOVA (η^2^ around 0.01, 0.06, and 0.14 are considered small, medium, and large, respectively).

Categorical analyses were carried out with the Chi-square test, with Yates correction whenever necessary.

## 3 RESULTS

The sample included N=590 professional workers. The detailed description of the sample is shown in Table 1. The sample included more women than men, except in Palestine (Table **1**). Participants were, on average, younger in Tunisia and Palestine than in Italy, with participants in Macedonia being in the middle (Table **1**).

Effect sizes, as measured by eta-squared, indicated that the most significant differences concerned the perception of the respect of users’ and staff’s human rights, with lower perceived respect of users’ human rights among participants from Palestine (η2= 0.101) and lower perceived respect of staff’s human rights among participants from Tunisia (η2 = 0. 233).

There were also differences across the countries concerning the perception of the impact of available resources on the effectiveness of care (η2 = 0.106). Participants from Italy and Palestine were those less concerned about the impact of lack of resources on the efficacy of care, while those from Tunisia and Macedonia were those most concerned. Overall, participants were less satisfied with the availability of resources for care that they were about other topics, with scores that were about a half as far as availability for resources was concerned than for different topic (Table **2** for details).

**Fig. (1):**
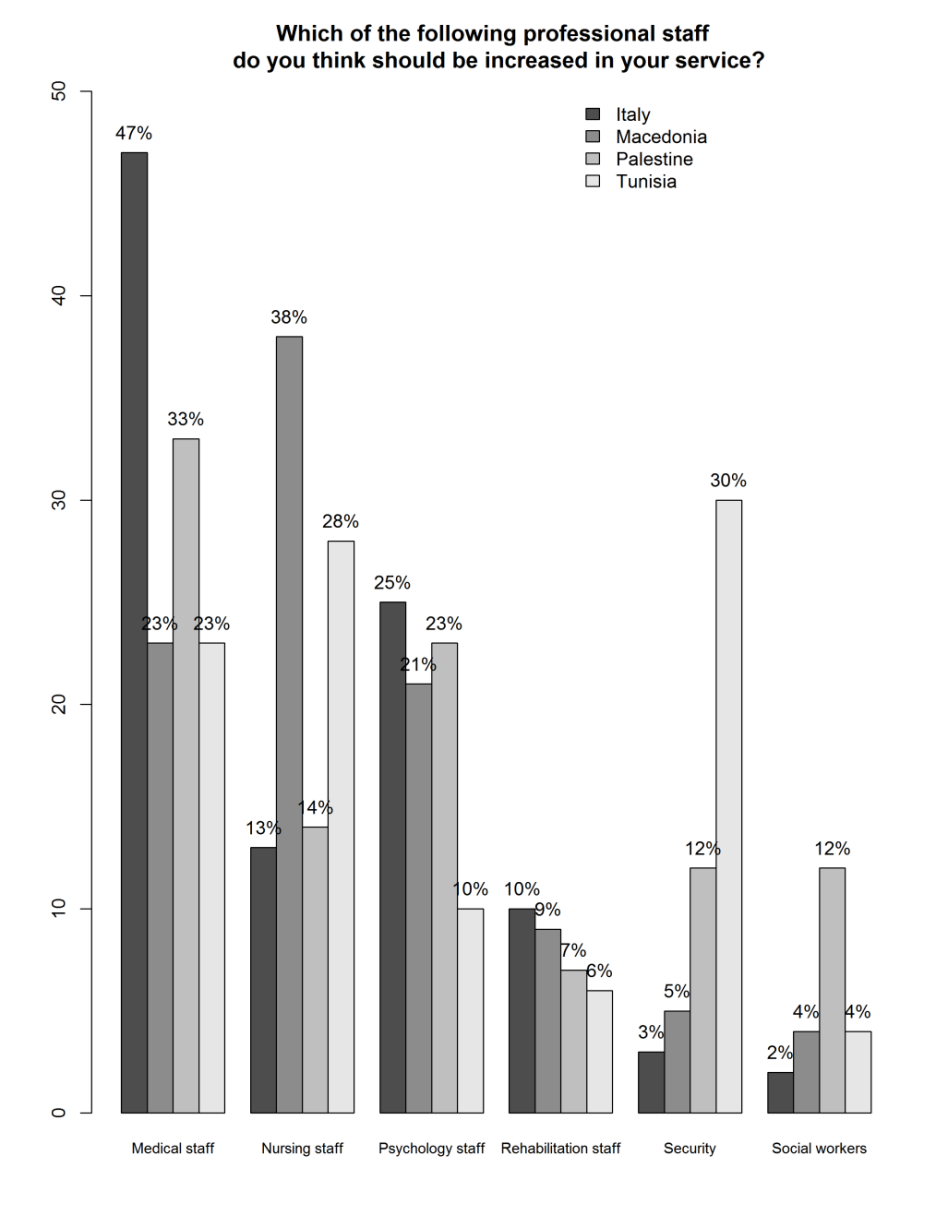
Distribution of scores on interventions required to improve resources by country.

There was also a difference in the study participants’ opinions on how to improve resources, with the suggestion to increase medical and psychology staff being the most often endorsed option (χ2 = 127.8; df = 18; p < 0.0001). However, participants from Tunisia and also from Palestine, albeit with lesser frequency, suggested to increase security staff as an intervention to improve resources (Fig. **1**).

## 4 DISCUSSION

The results of the distribution of the answers in the four samples showed the Italian staff as the most optimistic regarding work satisfaction, the perception of users’ satisfaction, as well as the perception of the respect of the Human rights of users. As for the organization of the services, the highest score was in Palestine with Italy and Macedonia, showing quite similar results in contrast to Tunisia that showed a much lower score. In the item dealing with the respect for the staff human rights, the higher score was in Macedonia with Tunisia achieving a remarkably low score in this item. In all four countries, the staff would like to have more doctors and psychologists present. In addition, participants from Tunisia and also from Palestine, albeit with lesser frequency, suggested to increase the number of security staff as an intervention to improve resources.

The set of results is very complex; hence interpretation of our findings is not easy.

On the basis of the general documents related to the available resources and the organization of the services in the four countries [5, 12-14] it is clear that the available resources in the Italian community services are much more consistent and more diversified than the resources in Macedonia, Tunisia, or Palestine. Moreover, the healthcare system in these countries is still very concentrated around hospital care. However, it is surprising that, although resources are probably more important in Italy than in other countries, staff still requires a larger number of doctors, psychologists and, to a lesser extent, of rehabilitation staff (such as Occupational Therapists, Educators or Rehabilitation Technicians). This likely reflects the emphasis in Italy on a healthcare model focused on medical treatment, rather than on an integrative approach [15]. In addition, in the region of Sardinia, where the study was conducted, a recent objective evaluation of staff numbers shows that there is an insufficient number of psychologists and rehabilitation workers [5].

It is important to note that in the Italian setting, there was a modest request for social workers, which is likely a reflection of the Italian community psychiatric services being oriented towards treatment (which requires different kinds of therapy and rehabilitation protocols) rather than towards assistance and care (which require interventions aimed at improving quality of life of the patients and their families).

However, despite the non-optimal availability of resources (at least on the basis of the aforementioned documents), the Palestinian sample, in comparison with the mean score of the other country samples, appears to be satisfied with his work, with the organization at work, and of the work in relationship with the available resources. However, the Palestinian sample scored the lowest in the perception of the respect of the users’ human rights. The doubt remains whether this is the result of actual reality of not respecting the rights or whether this is instead the consequence of a greater sensitivity of the staff and of their aspiration for improvement, or probably both.

In this context, it is difficult to interpret the result that requires greater number of security staff.

The sample of North-Macedonia is characterized by its low satisfaction with respect to work and its low satisfaction with available resources. However, they perceive that the rights of users and staff are respected and that they show themselves satisfied with the organizational aspects of their work. In essence, the professionals of North Macedonia seem dissatisfied above all with available resources.

The presence of a direct link between the available resources and the perception of the respect for human rights, although described in the validation work as a correlation between the respective elements [8], is not strong enough to appear clearly in North Macedonia. Indeed, the professionals in North Macedonia were dissatisfied with the adequacy of resources but relatively satisfied with the respect of human rights.

The Tunisian sample is characterized by, on average, lower scores than other nations, with markedly low results in organizational satisfaction, in the perception of the adequacy of resources, and in the perception of respect for the rights of the staff. A perception of insecurity is highlighted by a high percentage of professionals who consider it essential to increase the number of security staff. Indeed, the Tunisian sample scored very differently from the Italian sample. This may be attributed to a different organization of the healthcare system in Tunisia compared to Italy and also to the specific political situation that is currently running in Tunisia. The validation of the instrument had highlighted how, in the internal comparison between items within the same Tunisian sample, this low score did not correlate negatively with the scores of the other items. In particular, it was not negatively associated with respect for users' rights or access to resources [8]. For this reason, the result was interpreted as in relation to a general dissatisfaction of healthcare professionals in a particular historical moment of history, following the 2011 Revolution. The high number of staff that emphasized the importance of increasing security measures could be both the result of a perception of danger towards potential internal aggressors and the perception of vulnerability with respect to possible external aggressions. After the Revolution in 2011, aggressions on the staff in Tunisian hospitals have multiplied. One of the reasons might be that people are not yet able to sufficiently integrate the concept of individual freedom and rights, which they acquired after the Revolution. Overall, the Tunisian sample may be dissatisfied with the organization of their healthcare system for reasons that are unrelated to the topic that were investigated by the WWRR and that impact on the satisfaction at work for factors that impact on the respect of the human rights of the staff but less so on the respect of the human rights of the patients accessing the services.

## CONCLUSION

Our findings offer a useful insight into the perception of the quality of mental health services, especially from a users’ rights point of view. From this perspective, this type of research could help to formulate hypotheses about the way cultural differences and work context impact the perception of respect for users’ rights. This could stimulate the discussion and the implementation of projects allowing eventually to assess the validity of the hypotheses. However, the current study was cross-sectional, and no specific chain of causation could have been addressed in the exploration of the topics that were covered by the questionnaire. In the future, there is a need for replication studies accounting also for the actual resource utilization and including a longitudinal record of actual measures and to take into account the variables that might influence their perception of the respect of human rights of the patients and the staff.

## Figures and Tables

**Table 1:** General characteristics of the participants

-	Italy	Macedonia	Palestine	Tunisia	Chi-square
-	N = 126	N = 100	N = 164	N = 200	-
Men Women	45 (36%) 81 (64%)	39 (39%) 61 (61%)	88 (54%) 76 (46%)	78 (39%) 122 (61%)	χ^2^ = 12.2; df = 3; p = 0.007
<20 years old 20-29 years old 30-39 years old 40-49 years old 50-59 years old >60 years old	0 (0%) 0 (0%) 2 (2%) 30 (24%) 74 (59%) 20 (16%)	4 (4%) 21 (21%) 19 (19%) 28 (28%) 22 (22%) 6 (6%)	0 (0%) 28 (17%) 53 (32%) 46 (28%) 34 (21%) 3 (2%)	1 (0.5%) 60 (30%) 79 (39.5%) 30 (15%) 29 (14.5%) 1 (0.5%)	χ^2^ = 211.3; df = 15; p < 0.0001

The assessment tool was reliable, when measured with the Cronbach’s alpha, with partial exception of the scores from Tunisia: Italy = 0.83 (95%CI: 0.78 – 0.87); Macedonia = 0.99 (0.98 – 0.99); Palestine = 0.86 (0.82 – 0.89); Tunisia = 0.54 (0.44 – 0.64).

The four countries showed statistically significant differences with regards to the quality rights assessment tool (Table **2**).

**Table 2:** Comparison by country of scores on the quality rights assessment tool

All data: Mean (Standard deviation)	Italy	Macedonia	Palestine	Tunisia	ANOVA	Effect size
-	N = 126	N = 100	N = 164	N = 200	-	-
Staff’s satisfaction with job	4.1 (1.1)^a^	3.4 (1.4)^b^	3.7 (1.3)^b,c^	3.9 (1.6)^a,c^	F(3;586)=5.80, p=0.001	η^2^= 0.029
Users’ satisfaction with care	4.3 (1.0)^a^	3.9 (1.3)^a,b^	3.9 (1.3)^b^	3.7 (1.6)^b^	F(3;586)=4.71, p=0.003	η^2^= 0.024
Staff’s satisfaction with organization	3.6 (1.2)^a^	3.6 (1.3)^a^	3.7 (1.3)^a^	3.1 (1.6)^b^	F(3;586)=6.57, p<0.0001	η^2^= 0.033
Respect of users’ human rights	4.9 (1.2)^a^	4.2 (1.3)^b,d^	3.6 (1.5)^c^	3.8 (1.7)^c,d^	F(3;586)=21.9, p<0.0001	η^2^= 0.101
Respect of staff’s human rights	4.1 (1.2)^a^	4.3 (1.4)^a^	4.0 (1.4)^a^	2.4 (1.6)^b^	F(3;586)=59.4, p<0.0001	η^2^= 0.233
Staff’s satisfaction with resources for care	2.6 (0.8)^a^	2.0 (1.6)^b^	2.9 (1.3)^a^	2.1 (0.9)^b^	F(3;586)=23.2, p<0.0001	η^2^= 0.106

^a,b,c,d^ Games-Howell p<0.05; groups with the same superscript letter do not differ from each other

Participants from Italy reported, on average, the highest scores across the questions.
